# Turkish version of the pediatric quality of life inventory (PedsQL) 3.0 arthritis module: a reliability and validity study

**DOI:** 10.1186/1546-0096-9-S1-P140

**Published:** 2011-09-14

**Authors:** Tarakci Ela, Baydogan Nilay, Kasapcopur Ozgur, Dirican Ahmet

## Background and aim

Most research on health-related quality of life (HRQoL) to date has focused on adults. However, the need to address QoL issues in chronically ill children has become a priority. The measuring of HRQoL, especially for these chronically ill children, is of importance not only for describing their actual situation and their health care needs, but also for evaluating clinical interventions. The Pediatric Quality of Life Inventory builds on a programmatic measurement instrument development effort by Varni and colleagues in pediatric chronic health conditions, including rheumatic diseases during the past 20 years. The PedsQL 3.0 Arthritis Module was designed to measure HRQoL dimensions specifically tailored for pediatric rheumatology. Validated version of PedsQL 3.0 Arthritis Module has been published for use in other languages but there was no Turkish version. The aim of this article is to describe the translation, cultural adaptation and validation of a Turkish version of the PedsQL 3.0 arthritis module in a population of juvenile idiopathic arthritis (JIA).

## Methods

Total of 135 patients and 169 parents were enrolled in the study. The internal consistency reliabilities of the PedsQL 3.0 Arthritis Module scales generally exceeded the recommended minimum alpha coefficient standard of 0.70 for group comparisons for self report by children ages 8–18 and for parent proxy report for children ages 2–18.

## Results

Total score for parent Proxy report for children ages 2–18 and self report by children ages 5-18 had alpha coefficients of 0.56–0.84. Test-retest reliability was indicated by the highly significant correlation for ages groups PedsQL parent’s reports and patient’s self reports. When subscales correlations were evaluated and above indicated that the scale was highly reliable.

## Conclusion

The results of this study show that the PedsQl 3.0 Arthritis Module is suitable for long-term longitudinal clinical and epidemiologic studies, and for comparing children of different ages with Turkish JIA .

